# Protocol for generating a 3D hydrogel-based tumor resection model *in vitro* using pancreatic cancer cells

**DOI:** 10.1016/j.xpro.2026.104416

**Published:** 2026-03-05

**Authors:** Lea Miebach, Marten Hagge, Linus Hübner, Sander Bekeschus

**Affiliations:** 1 ZIK Plasmatis, Leibniz Institute for Plasma Science and Technology (INP), Felix-Hausdorff-Street 2, 17489 Greifswald, Germany; 2 Department of Hematology and Oncology, Greifswald University Medical Center, Sauerbruchstr., 17475 Greifswald, Germany; 3 Department of General, Visceral, Thoracic, and Vascular Surgery, Greifswald University, Medical Center, Ferdinand-Sauerbruch-Street, 17475 Greifswald, Germany; 4 Department of Dermatology, Venerology, and Allergology, Rostock University Medical Center, Strempelstr. 13, 18057 Rostock, Germany

**Keywords:** cell culture, cancer, health sciences, microscopy, organoids

## Abstract

We present a protocol for the generation of two distinct *in vitro* tumor resection models to evaluate cellular responses at tumor margins in 3D. We describe steps for patterning pancreatic cancer cells (Panc-01) embedded in hydrogels using a custom 96-pin metal lid to create standardized resection cavities. In a second model, we detail procedures for refining the protocol to imitate narrow surgical resection margins. This protocol supports diverse treatment modalities and enables reproducible, high-throughput analysis of post-resection responses.

For complete details on the use and execution of this protocol, please refer to Miebach et al.^1^

## Highlights

• Protocol for generating standardized 3D tumor resection cavities in hydrogel
• Instructions for embedding tumor cells and creating defined resection margins
• Guidance on applying chemical, oxidative, or physical post-resection treatments
• Steps for viability assays and live imaging with automated margin-specific analysis

## Abstract (editor highlights)

Publisher’s note: Undertaking any experimental protocol requires adherence to local institutional guidelines for laboratory safety and ethics.

## Graphical abstract

**Figure:**
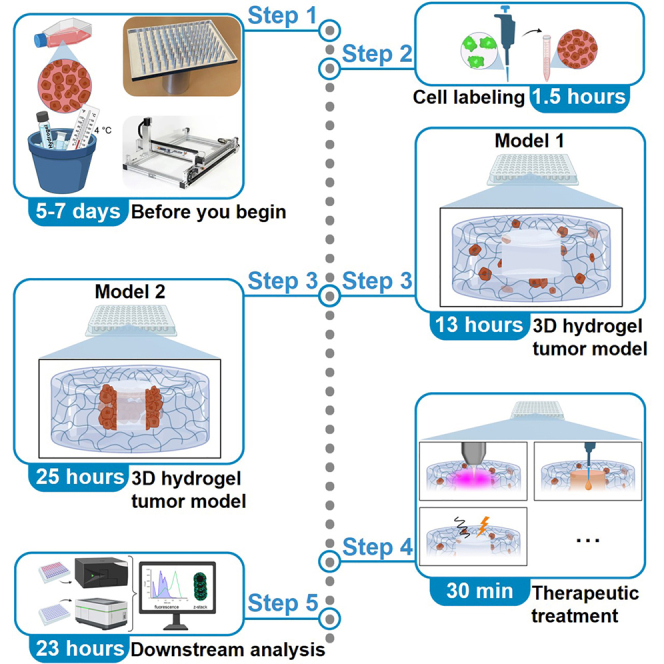

## Before you begin

**Figure 1:**
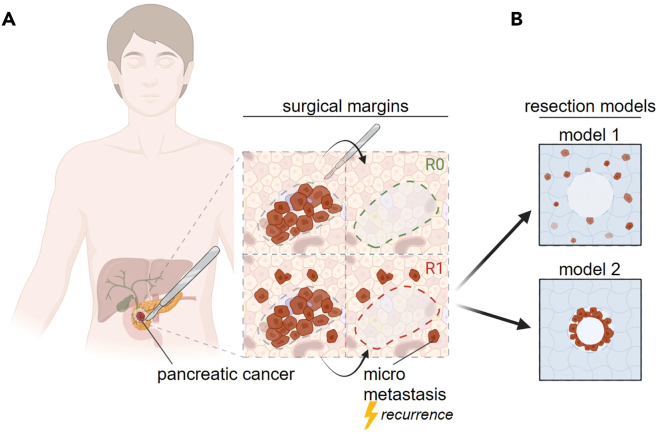
Schematic of tumor resection margins (A) surgical margins illustrating R0 and R1 micro metastatic conditions; (B) R1 resection models outlined in this study. Figure reprinted and adapted with permission from Miebach et al., 2025.

Tumor recurrence following surgical resection remains a major clinical challenge, often resulting from residual malignant cells at the surgical margin (Figure 1A).^2^^,^^3^^,^^4^ Understanding how these cells respond to different post-resection treatments is essential for developing strategies that enhance local tumor control and improve therapeutic outcomes. Traditional in vitro assays and two-dimensional culture systems are limited in their ability to replicate the complex, three-dimensional structure and cellular organization of tumor tissue.^5^^,^^6^ To address this, we established standardized 3D in vitro R1 tumor resection models that simulate the physical and biological context of a surgical wound within a tumor-like microenvironment. By embedding tumor cells within a hydrogel matrix and generating defined resection cavities using a custom metal pin array, this system provides a reproducible framework for analyzing treatment-induced effects, such as cell death, migration, or recovery, at and around the resection site. An alternative protocol increases complexity by establishing a layered tumor-stroma architecture in which a defined tumor core is embedded in a cell-free matrix before simulated resection. This version mimics more aggressively growing tumor cells in the periphery and narrow resection margins (Figure 1B).

Both, model 1 and 2 can principally accommodate a range of post-resection interventions, including chemical, oxidative, thermal, or physical treatments, thereby serving as flexible tools for studying tumor margin responses under controlled laboratory conditions. In combination with high-content imaging and automated segmentation analysis, both models enable precise quantification of treatment effects as a function of distance from the simulated surgical margin.

***Note:*** Both models described in this protocol follow similar overall procedures. The distinction lies in how the tumor resection model is configured to address specific experimental questions. Select the configuration that best matches your intended treatment scenario or biological hypothesis.

### Institutional permissions

Not applicable, this study did not involve animal experiments or human trials.

**Figure 2:**
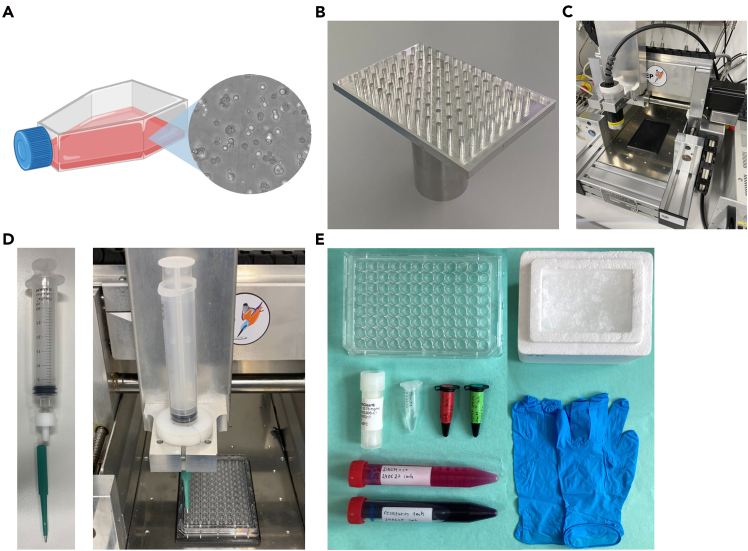
Overview of equipment required for this protocol (A) T-75 flask with tumor cells; (B) 96-pin punch plate; (C) xyz positioning table; (D) biopsy punch setup; (E) staining materials. Figure reprinted and adapted with permission from Miebach et al., 2025.

### Biological and chemical setup: Cell culture and hydrogel preparation

**Timing: 5–7 days**

***Note:*** The outlined protocol is optimized for the human pancreatic cancer cell line Panc01 but can be adapted to other cell lines that are of interest. Be aware that tumors with distinct growth patterns, such as highly invasive gliomas, desmoplastic tumors, or non-epithelial malignancies, may exhibit variations in adhesion, migration, and matrix remodeling that can affect cavity stability and readouts. We recommend preliminary testing and optimization of cell density, hydrogel composition, and incubation parameters when using alternative cell lines.

***Note:*** This protocol can be adapted to physiologically more relevant conditions, including co-culture with fibroblasts or macrophages, incorporation of patient-derived tumor organoid fragments, or integration of endothelial-like networks.

***Note:*** The duration of this step depends on the growth rate of the cell line used.

***Note:*** Panc01 cells are maintained in Dulbecco’s Modified Eagle Medium (DMEM) supplemented with 10% FCS, 1% penicillin/streptomycin, and 1% L-glutamine. Alternative media compatible with your specific cell line may be used if they maintain healthy growth and viability.

1. Thaw and cultivate cells following cell line-specific requirements (Figure 2A).
  a. Monitor cells daily and subculture once they reach approximately 80% confluence (see problem 1).
  b. Discard the cell culture medium and gently rinse the flask once with 10 mL sterile PBS.
  c. Detach cells using 3 mL/75cm^2^ Accutase and incubate for 15 min at 37°C.
    **CRITICAL:** Use Accutase instead of trypsin to ensure gentle detachment. Overly harsh enzymatic or mechanical dissociation can damage membranes and affect downstream 3D culture integrity and viability.
  d. Confirm detachment of cells under the microscope.
  e. Rinse the flask once with 5 mL PBS to collect any remaining cells.
  f. Transfer the cell suspension into a 15 mL conical tube and centrifuge at 300 × *g* for 5 min.
  g. Discard the supernatant carefully and resuspend the cell pellet in 5 mL sterile PBS.
  h. Determine cell count and viability using flow cytometry with DAPI staining to discriminate dead cells.
    ***Note:*** Alternative cell counting methods, such as hemocytometer counting with trypan blue or automated counters, can be used if they provide accurate viability assessment. For subculturing, transfer 1 × 10^6^ viable cells into a T75 flask.
  i. Add 10 mL of complete DMEM and incubate under standard conditions.
    ***Note:*** Maintain consistent subculturing ratios to ensure reproducible growth characteristics, viability, and treatment responses. Cells should be maintained in standard culture for about 1 week after thawing before use.
2. Prepare the hydrogel.
  **CRITICAL:** We strongly recommend using commercially manufactured and mechanically rigid hydrogels, as they provide more reliable consistency, structural stability, reproducibility, and cell viability in this setup. Be aware that batch-to-batch variations in stiffness, porosity, and diffusion properties can impact tumor cell behavior and treatment efficacy. Young’s modulus reference values for hydrogel stiffness and diffusion ranges of small molecules can guide adjustments for different matrices or laboratories. Users wishing to explore alternative hydrogels, such as collagen or agarose, should consider performing mechanical characterization and optimization to maintain cavity integrity.
  ***Note:*** Because hydrogel begins to polymerize at temperatures above 15°C, always handle it on ice to prevent premature polymerization (see problem 2).
  a. Thaw the hydrogel on ice.
    ***Note:*** Pre-cool tubes before handling hydrogel to prevent premature polymerization.
  b. Mix gently by swirling or use a pipette.
    ***Note:*** Avoid introducing air bubbles.
  c. Aliquot hydrogel into pre-chilled tubes.
    ***Note:*** For 3D tumor model formation, you need 150 μL of hydrogel per well. Adjust aliquot volumes as needed for experimental design.

### Mechanical setup: Prepare the 96-pin punch plate, xyz table, and biopsy punch

**Timing: 4 h (excluding fabrication time)**

This step describes the preparation and handling of the custom metal lid equipped with 96 evenly spaced pins used to generate standardized resection cavities in the 3D tumor model (Figure 2B), as well as the configuration of the computer-controlled xyz table used for precise and contact-free lid removal (Figure 2C).

3. Fabricate or obtain the 96-pin punch plate.
  ***Note:*** The plate can be fabricated in-house or outsourced according to the specified dimensions.
  a. Clean and sterilize the punch plate prior to experiments using disinfectants.
    ***Note:*** Handle the lid only with clean gloves to maintain sterility.
4. Set up and calibrate the xyz table for lid removal.
  ***Note:*** Use a computer-controlled, motorized xyz stage to ensure standardized cavity formation. We do not recommend manual removal, as it showed significant variation across experiments and may cause detachment of the gel or loss of defined cavity geometry. If manual removal has to be performed instead of automated lifting, use a consistent and slow upward motion to minimize mechanical stress on the matrix.
  a. Calibrate the xyz table by setting zero coordinates at the upper edge of the plate and the top plane of the metal lid.
  b. Define a precise z-axis movement to lift the metal lid vertically without lateral displacement.
  c. Program moderate movement speeds to ensure gentle detachment and avoid disrupting the 3D matrix structure.
    ***Note:*** We recommend testing the movement without samples to confirm smooth, uniform operation before proceeding to experiments.
5. Test the setup for clamping the 3 mm biopsy punch into the xyz table (Figure 2D).
  a. Secure a sterile 10 mL syringe in the xyz table holder.
  b. Insert the 3 mm biopsy punch into the syringe adapter.

***Note:*** Calibration of the xyz table may require adjustment to ensure that the punch precisely targets the intended location. Test the movement before proceeding with the experimental samples.

## Key resources table

**Table:** 

REAGENT or RESOURCE	SOURCE	IDENTIFIER
**Chemicals, peptides, and recombinant proteins**		

Dulbecco’s Modified Eagle Medium (DMEM)	Pan Biotec	Cat#F0445
Fetal bovine serum	Pan Biotec	Cat# P40-1401
Penicillin-streptomycin	Pan Biotec	Cat# P06-07100
L-glutamine	Pan Biotec	Cat# P04-80100
Phosphate buffered saline	Pan Biotec	Cat# P04-36500
Hydrogel	R&D Systems	Cat#3432-005-01
DAPI	Carl Roth	Cat#6335.1
Vybrant DiD Cell Labeling Solution	Thermo Fisher	Cat#V22887
SYTOX Green	Thermo Fisher	Cat#S7020

**Experimental models: Cell lines**		

Panc-1	ATCC	Cat#CRL-1469

**Software and algorithms**		

Harmony 4.9	PerkinElmer	N/A
BioRender	BioRender	RRID: SCR_018361

**Other**		

Breeding incubator	Binder	Cat#9010-0319
96-pin punch plate	Miebach et al.^1^	N/A
Motorized, computer-controlled xyz table	CNC	Cat#13110000
Biopsy punchout	KAI Europe	Cat#49301
Disposable syringe	BD	Cat#115256
Microplate reader	Tecan	Cat#30050303
High-content imaging system	PerkinElmer	Cat#HH16000000

## Materials and equipment

**Table:** Complete Dulbecco’s Modified Eagle Medium (DMEM)

Reagent	Final concentration	Amount
Dulbecco’s Modified Eagle Medium (DMEM)	88%	88 mL
Fetal bovine serum	10%	10 mL
Penicillin-streptomycin	100 U/mL - 100 μg/mL	1 mL
L-glutamine	2 mM	1 mL
**Total**	**N/A**	**100 mL**

The medium can be stored for 2–3 months at 4°C.

## Step-by-step method details

### Cell labeling

**Timing: 1.5 h**

This step describes the fluorescent labeling of tumor cells and outlines required equipment for the procedure (Figure 2E).

***Note:*** Alternative cell dyes compatible with your imaging system can be used if they provide sufficient contrast and stability.

1. Harvest the tumor cells at approximately 80% confluence and determine cell count as described above.
2. Adjust the concentration to obtain 3 × 10^5^ cells per 200 μL.

***Note:*** Adjust the cell concentration as needed when using other cell lines to achieve comparable cell density and growth characteristics.

3. Add 500 nM Vybrant DiD Cell labeling solution.
4. Incubate for 45 min at 37°C in the dark.

***Note:*** Avoid prolonged incubation to prevent reduced cell viability or uneven staining.

5. Wash the cells twice with PBS to remove excess dye.
6. Centrifuge at 300 × *g* for 5 min.
7. Discard supernatant.

***Note:*** For the following 3D tumor resection model formation steps, follow steps 8–13 to generate model 1, and steps 14–22 to generate model 2.

### 3D hydrogel tumor model 1

**Timing: 13 h**

This step describes the generation of a 3D tumor resection model and generation of standardized cavities using the custom 96-pin punch plate.

**Figure 3:**
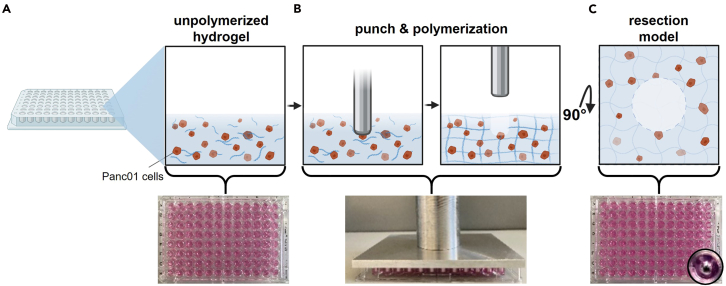
Steps for formation of the 3D hydrogel tumor resection model 1 (A) hydrogel containing Panc01 tumor cells; (B) introduction of the 96-pin punch plate and incubation at 37°C leads to polymerization of hydrogel; removal of 96-pin punch plate generates cavities mimicking R1 surgical margins; (C) top view of the resection and adjacent tumor cells representing peripheral micro metastases. Figure reprinted and adapted with permission from Miebach et al., 2025.

8. Resuspend the labeled cells in 150 μL hydrogel per 3 × 10^5^ cells on ice.
9. Add 1 μM of SYTOX green.
10. Transfer 200 μL cold hydrogel with 3 × 10^5^ labeled tumor cells per well into the cavities of the 96-well plate (Figure 3A).
11. Carefully place the pre-sterilized 96-pin metal lid on top of the plate, ensuring full contact with the hydrogel.
12. Incubate 12 h at 37°C, 5% CO_2_, and 95% humidity to allow gel polymerization and formation of resection cavities around the pins (Figure 3B).
13. Mount the plate on the computer-controlled xyz table and gently remove the metal lid to expose uniform cavities simulating tumor resection sites (Figure 3C) (see problem 3).

***Note:*** If you successfully generated tumor resection model 1, now directly proceed to step 23 for therapeutic/treatment delivery.

### 3D hydrogel tumor model 2

**Timing: 25 h**

These steps describe the generation of an alternative 3D tumor resection model by forming a two-layer construct to model narrow tumor resection margins.

**Figure 4:**
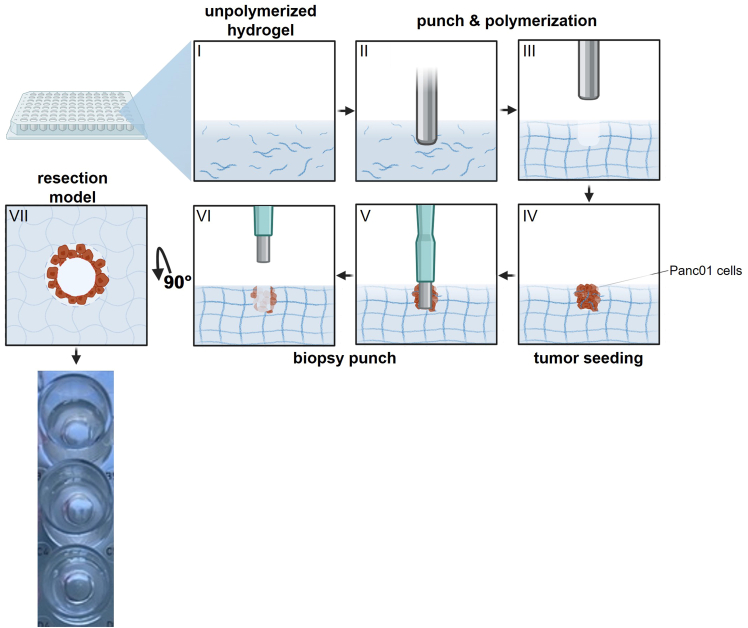
Steps for formation of the 3D hydrogel tumor resection model 2 96-well plate with: I: unpolymerized hydrogel, II: insertion of the punch plate, III: removal of the punch plate exposing tumor-seeding cavity IV: tumor seeding with Panc01 cells, V: insertion of 3 mm biopsy punch, VI: removal of 3 mm biopsy punch revealing resection site, VII: top view highlighting the narrow surgical margin and bordering tumor cells. Figure reprinted and adapted with permission from Miebach et al., 2025.

14. Add 140 μL cell-free hydrogel to each well (Figure 4 I).
15. Carefully place the pre-sterilized 96-pin metal lid on top of the plate, ensuring full contact with the hydrogel (Figure 4 II).
16. Incubate 12 h at 37°C, 5% CO_2_, and 95% humidity to allow gel polymerization and formation of resection cavities around the pins.
17. Mount the plate on the computer-controlled xyz table and gently remove the metal lid to expose uniform cavities around the pins (Figure 4 III).
18. Resuspend the labeled cells in 30 μL hydrogel per 2 × 10^5^ cells on ice.
19. Add 1 μM of SYTOX green.
20. Add 30 μL h containing 2 × 10^5^ DiD-labeled cells into the formed cavity to establish a defined tumor core (Figure 4 IV).
21. Allow the gel to solidify for 12 h at 37°C, 5% CO_2_, and 95% humidity.
22. Use the 3 mm biopsy punch, by continuous traction on the syringe plunger, to remove the tumor core (Figure 4 V, VI) and generate uniform cavities simulating tumor resection sites (Figure 4 VII).

### Therapeutic/treatment delivery

**Timing: 30 min**

**Figure 5:**
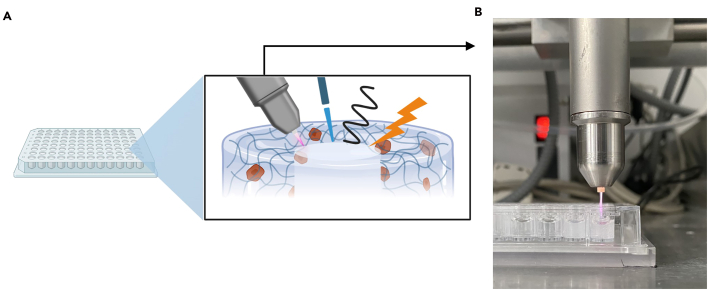
Treatment of the tumor resection cavities (A) overview of potential applications; (B) cold gas plasma treatment.

This step describes the application of experimental treatments to the simulated resection cavities (Figure 5A). Although exemplified here with cold gas plasma treatment (Figure 5B), both models support a variety of treatment modalities, including chemical, physical, or biological interventions, depending on the experimental objective.

23. Immediately after lid removal, apply the desired post-resection treatment to each cavity.

**CRITICAL:** Minimize delay between lid removal and treatment to prevent matrix dehydration and ensure consistent treatment exposure.

***Note:*** For oxidative or chemical treatments, pipette 30 μL of the treatment solution directly into the cavity. For physical treatments (e.g., cold gas plasma (Methods video S1), photodynamic therapy, etc.), follow device-specific parameters.

***Note:*** When using any treatment modality, ensure compliance with local safety and equipment handling guidelines.

24. Incubate or treat for defined period.
25. Carefully remove any residual treatment solution and replace with 30 μL fully supplemented DMEM.

### Downstream analysis

**Timing: 1.5–23 h (depending on sample size and analysis method)**

**Timing: 1.5 h (for step 26)**

**Timing: 23 h (for step 27)**

This section outlines downstream analytical approaches for quantifying treatment-induced effects in the 3D tumor resection models. The protocol primarily describes two complementary readouts: a resazurin-based metabolic viability assay and z-resolved high-content imaging for spatially resolved viability analysis. Both methods can be performed independently or in parallel to assess global metabolic activity and margin-specific cellular responses.

***Note:*** Although only these two readouts are detailed here, the models are compatible with additional downstream analyses depending on the conducted treatment and experimental objective. Furthermore, endpoint analyses are performed 1.5–23 h h post-treatment; however, extended imaging or assays at 48–72 h may provide additional insight into delayed cell regrowth or resistance mechanisms.

**Pause point:** Depending on your experimental design and analysis timeframe, treated cells can be maintained in complete DMEM for up to 48 h before proceeding with analyses. Ensure that cell viability is maintained and medium is refreshed as needed to prevent nutrient depletion.

**Figure 6:**
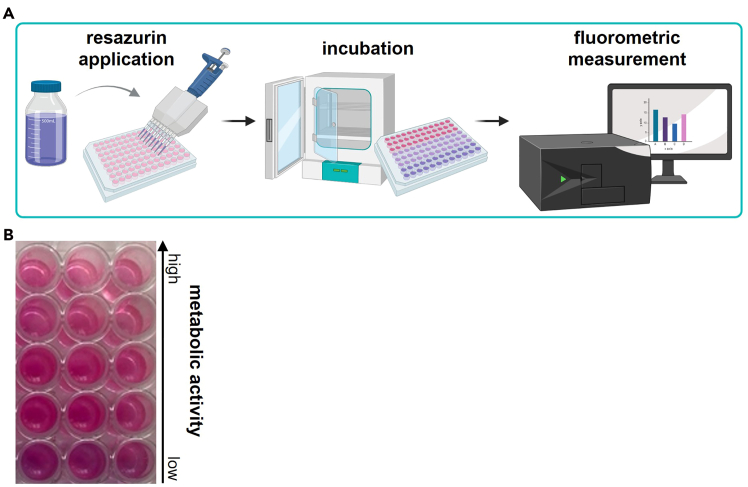
Resazurin-based metabolic viability assay (A) Overview of the general workflow; (B) example of resazurin-stained wells showing fluorescence readout indicative of cellular metabolic activity.

**Figure 7:**
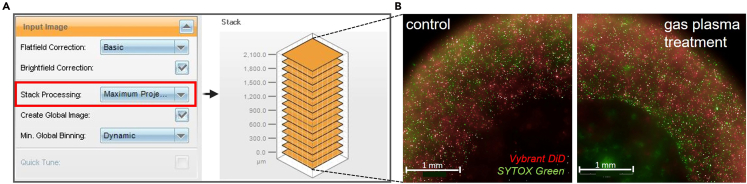
Representative imaging analysis results (A) image stack processing and generation of “Maximum Projection Images” in Harmony software; (B) example maximum-projection images from z-stacks acquired by high-content imaging of control and gas-plasma-treated tumor resection model 1 stained with Vybrant DiD and SYTOX Green. Figure reprinted and adapted with permission from Miebach et al., 2025.

**Figure 8:**
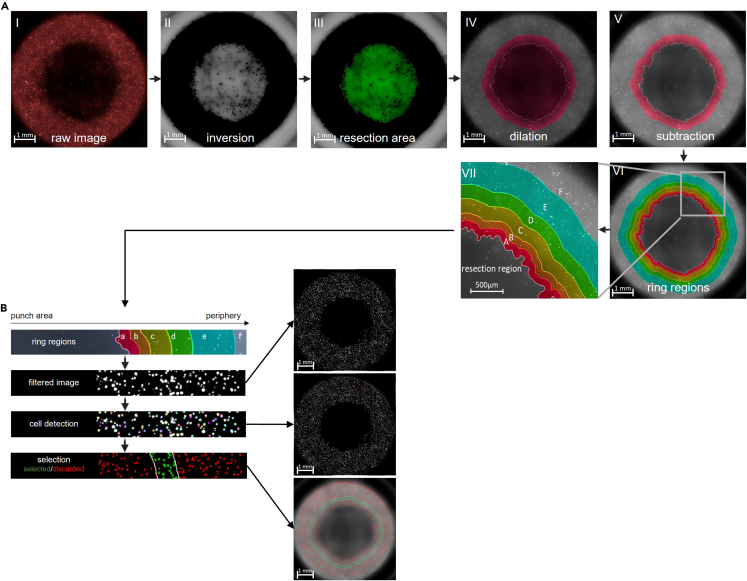
Algorithm-based automated picture processing and quantitative analysis with image analysis software (A) steps for determination of ring sections: I: raw image, II: inversion, III: algorithmically detected tumor resection region, IV: dilation of tumor resection region, V: subtraction of inner region VI: determination of ring regions, VII: magnification; (B) steps for cell detection and quantification. Figure reprinted and adapted with permission from Miebach et al., 2025.

26. Resazurin assay.
  This step describes a fluorescence-based viability assay using resazurin applied to the resection cavities. A fluorescence-capable plate reader is required for quantification (Figure 6A). The resulting fluorescence signals reflect cellular metabolic activity in each cavity (Figure 6B).
  a. Remove medium from wells.
  b. Add 30 μl resazurin (1 mM) into each cavity.
  c. Incubate plate at 37°C, 5% CO_2_ for 1 h protected from light.
  d. Measure fluorescence at λ_ex_ = 535 nm and λ_em_ = 590 nm.
27. Z-resolved imaging.
  This step details live-cell imaging and quantitative analysis of cell viability as a function of distance from the resection margin. A z-resolved high-content imaging system capable of live-cell imaging is required.
  a. Place the treated plate into a high-content imaging system.
  b. Capture time-lapse z-stack images with a plane-to-plane distance of 300 μm at multiple time points over a 20 h period using a 5× air objective (NA 0.16).
    ***Note:*** The number of z-stacks, step size, magnification, imaging intervals, and overall imaging duration can be adjusted to fit experimental needs, but must remain consistent across wells and replicates (see problem 4).
  c. Use algorithm-based image analysis to automatically:
    i. Generate maximum intensity projections of z-stacks (Figures 7A and 7B).
    ii. Invert the raw image (Figure 8A I, II).
    iii. Segment the resection area (Figure 8A III).
    iv. Dilate the resection area (Figure 8A VI).
    v. Subtract the resection area (Figure 8A V).
    vi. Perform image segmentation to define the resection margin and concentric ring regions extending outward (e.g., 0–100 μm, 100–200 μm, 200–400 μm, 400–600 μm, 600–1000 μm, and 1000 μm-well border) (Figure 8A VI, VII).
    vii. Quantify live and dead cells based on fluorescence intensity thresholds (Figure 8B) (see problem 5).
      ***Note:*** Not all analytical procedures are described in full detail. High-content image analysis is inherently complex and depends on experiment-specific conditions. Adapt analysis steps and parameter settings to the requirements of your experiment and anticipate the need to test several variants to optimize the analysis procedure.
      ***Note:*** Other image analysis platforms (e.g., ImageJ / Fiji, CellProfiler) can be used for image segmentation.

## Expected outcomes

This protocol yields a reproducible three-dimensional in vitro model that mimics tumor resection margins. When executed properly, the hydrogel matrix forms stable hemispherical cavities with well-defined edges after removal of the metal pin array. In the model 1 configuration, tumor cells are uniformly distributed within the hydrogel, whereas the model 2 setup with an embedded tumor core models narrow surgical margins.

Following a variety of potential treatment applications, spatially resolved imaging reveals gradients of cell viability that can be quantified across defined distance zones from the simulated resection cavity. This setup enables high-throughput evaluation of local treatment effects, margin-specific cytotoxicity, and potential regenerative responses. Taken together, this protocol supports studies on local treatment penetration and could, in general, be extended to analyze treatment-induced alterations in cellular behavior.

## Limitations

While this protocol provides a robust and standardized approach for modeling tumor resections in vitro, several limitations should be considered. First, the models represent simplified tumor environments composed of a single cell type and lack stromal, vascular, or immune components that influence tumor behavior in vivo. Furthermore, biological hydrogel matrices, although well-suited for this setup, may exhibit batch-to-batch variability in composition and stiffness, which can affect reproducibility.

Additionally, the standardized resection geometry does not fully capture the structural complexity or biochemical heterogeneity of real tumor margins. Diffusion and penetration of applied treatments may differ from in vivo conditions due to the absence of tissue perfusion and dynamic interstitial flow. Therefore, while highly reproducible and well-suited for comparative studies, these models, like any other, should be regarded as a reductionist system for assessing localized post-resection treatment responses under controlled conditions.

## Troubleshooting

### Problem 1: Poor cell cultivation performance

There are many potential issues that can occur during cell cultivation, with perhaps equally as many underlying causes. These problems may manifest as reduced proliferation, abnormal morphology, or inconsistent treatment responses. If such difficulties arise, the general troubleshooting approaches listed below may help guide you toward identifying and correcting the issue. For more specific troubleshooting strategies, refer to established cell cultivation protocols or the manufacturer’s cell line documentation.

### Potential solution

Ensure sterility: Maintain strict technique during all culture manipulations. Regularly clean incubators and workspaces to minimize contamination risk.

Optimize passage timing: Subculture cells at 70%–80% confluence to prevent overgrowth, nutrient depletion, or contact inhibition. Avoid leaving cells confluent for extended periods.

Verify culture conditions: Confirm that temperature, CO_2_ concentration, and humidity are stable and appropriate for the specific cell line.

Use high-quality media and supplements: Prepare media fresh, use validated serum lots, and ensure additives are not expired.

Control cell line quality: Use low-passage, authenticated, and mycoplasma-free cells. Routinely monitor morphology and proliferation rates.

Avoid handling stress: reduce centrifugation steps, pipetting, or exposure to non-ideal temperatures, if possible. Handle cells gently during detachment and seeding.

Record and standardize: Keep detailed records of passage numbers, media batches, and environmental parameters to ensure reproducibility across experiments.

### Problem 2: Premature polymerization of hydrogel

Most hydrogels rapidly solidify above 10°C–15°C, which can lead to inconsistent gelation or air bubble formation during handling.

### Potential solution

Handle the hydrogel on ice at all times and pre-cool tubes and plates before dispensing. Work swiftly to prevent local warming. If polymerization occurs before seeding, discard the mix or affected wells and prepare fresh hydrogel.

### Problem 3: Inadequate or irregular resection cavities

**Figure 9:**
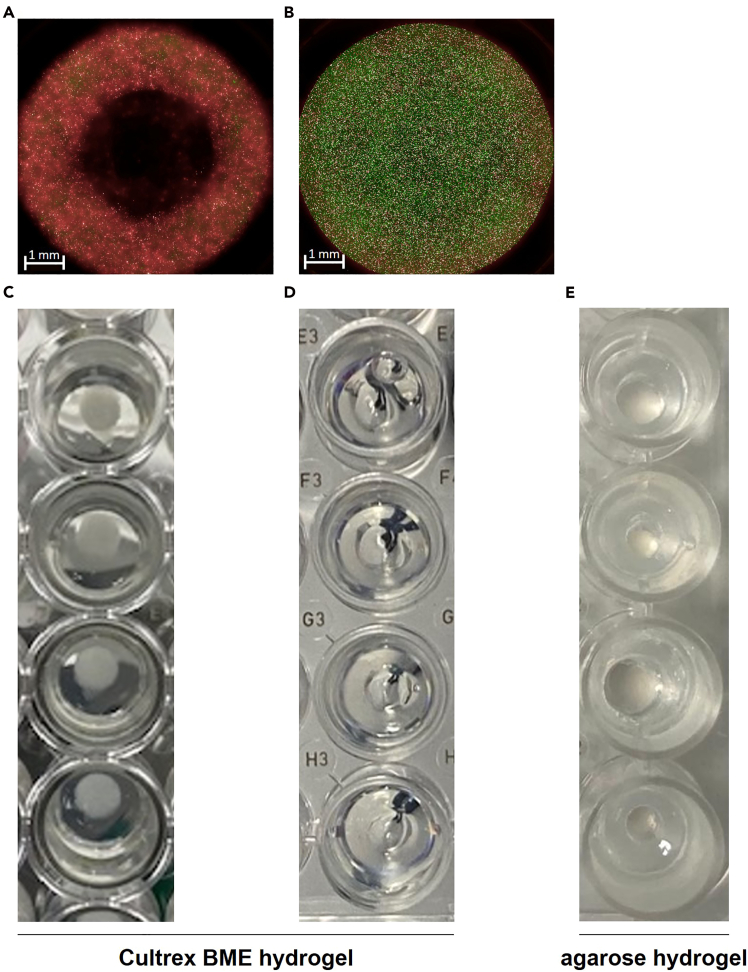
Representative examples of cavity formation outcomes in hydrogel (A and C) desired outcomes; (B and D) undesired outcomes; (E) tumor resection model using agarose hydrogel.

Insufficient or uneven cavity shapes (Figures 9A–9D) may result from poor contact between the metal pin and the hydrogel surface, incomplete gel polymerization, or debris on the pins. Additionally, biologically derived hydrogels show batch-to-batch variability, which may influence gel consistency and cavity formation. Note that for self-prepared gels, e.g., agarose or collagen hydrogels, we experienced significant problems with polymerization, uniformity, and rigidity of resection cavities (Figure 9E).

### Potential solution

As stated before, we highly recommend using commercially manufactured hydrogels with sufficient mechanical rigidity. If problems persist, ensure that the 96-pin metal lid is correctly aligned and maintains uniform contact with all wells during incubation. Clean and sterilize the pins thoroughly before each use to prevent mechanical obstruction. After incubation, verify that the hydrogel has fully polymerized before removing the lid to maintain cavity integrity. If irregularities continue across experiments, consider switching to a fresh batch of hydrogel or to a hydrogel from another manufacturer.

### Problem 4: Inconsistent or weak fluorescence staining

**Figure 10:**
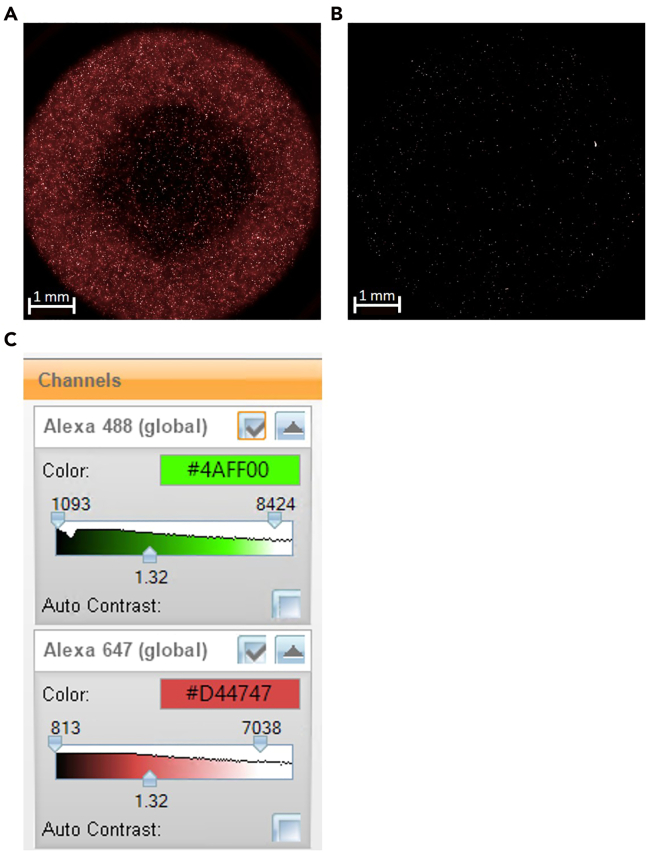
Representative examples of fluorescence staining outcomes (A) desired fluorescence signals; (B) undesired fluorescence signals; (C) regulation of fluorescence thresholding in Harmony software.

Variability in fluorescence intensity may arise from inaccurate dye preparation, insufficient mixing, or uneven washing (Figures 10A and 10B). Furthermore, fluorescence threshold and contrast may require adjustment (Figure 10C).

### Potential solution

Verify the dye concentration, incubation time, and cell density. Mix samples gently but thoroughly to ensure uniform labeling. After staining, wash carefully to remove unbound dye and minimize background fluorescence. Protect samples from light throughout the procedure. Additionally, assess fluorescence intensities and adjust acquisition parameters as needed.

### Problem 5: Poor segmentation or inconsistent quantification during image analysis

Incorrect thresholding or background signal can reduce segmentation accuracy and data comparability.

### Potential solution

Optimize thresholding parameters for each fluorophore. Validate segmentation with representative control wells before batch processing. Maintain consistent region definitions across all samples for comparative analyses.

## Resource availability

### Lead contact

Further information and requests for resources and reagents should be directed to and will be fulfilled by the lead contact, Sander Bekeschus (sander.bekeschus@med.uni-rostock.de).

### Technical contact

For questions regarding the technical execution of this protocol, please contact the technical lead, Lea Miebach (leakatharina.miebach@med.uni-greifswald.de), who can provide guidance and support.

### Materials availability

This study generated a custom-made 96-pin punch plate. The graphic file for the template is available upon request.

### Data and code availability

• All data are available from the lead author upon request.
• This article does not report original code.
• Any additional information required to reanalyze the data reported in this article is available from the lead contact upon request.

## Acknowledgments

The graphical abstract and figures were created using Biorender.com. Technical support from Felix Niessner and Henry Skowski is gratefully acknowledged. This work was funded by the German Federal Ministry of Education and Research (BMBF; grant nos. 03Z22DN11 and 03Z22Di1 to S.B.) and the Gerhard-Domagk (Greifswald, Germany) scholarship to M.H. The funding sources had no role in the design of this study or its execution, analyses, interpretation of the data, or the decision to publish results.

## Author contributions

Conceptualization, S.B. and L.M.; investigation, L.M. and M.H.; writing – original draft, L.H.; writing – review and editing, S.B., L.H., and L.M.; funding acquisition, S.B.; supervision, S.B.

## Declaration of interests

The authors declare no competing interests.

## Supplemental information

Methods video S1. Cold gas plasma treatment of the resection cavity with the kINPen, related to step 23
